# Inclusion of men in maternal and safe motherhood services in inner-city communities in Ghana: evidence from a descriptive cross-sectional survey

**DOI:** 10.1186/s12884-017-1590-3

**Published:** 2017-12-14

**Authors:** Margaret Duah Atuahene, Sylvia Arde-Acquah, Nana Frema Atuahene, Martin Adjuik, John Kuumuori Ganle

**Affiliations:** 1 Department of Population, Family and Reproductive Health, School of Public Health, University of Ghana, P.O. Box LG 13, Legon, Accra, Ghana; 2 School of Public Health, University of Ghana, Accra, Ghana; 3 Augusta Medical Centre, Waynesboro, Virginia USA; 4 INDEPTH Network Secretariat, Accra, Ghana

**Keywords:** Men inclusion, Maternal health services, Safe motherhood, Family planning, Spousal communication, Survey, Inner-city communities, Ghana

## Abstract

**Background:**

There has been a growing realisation of the need to enhance men’s inclusion in maternal and safe motherhood services, especially in low-income settings. However, empirical studies on the extent to which men are involved in maternal and safe motherhood services especially in poor inner-city communities are lacking. The purpose of this study was to describe the level of men’s inclusion in maternal and safe-motherhood services in inner-city communities in Ghana, and to assess the barriers of men’s involvement.

**Methods:**

A descriptive cross-sectional quantitative survey was conducted among a total of 256 randomly selected adult men in Chorkor, an inner-city fishing community in Accra, the capital city of Ghana. A multistage sampling strategy was used to select houses, households and respondents. Descriptive statistical techniques were used to analyse the data. Data analysis was done with the aid of SPSS version 20.

**Results:**

Although almost all (96.6%) respondents knew the meaning of family planning, as high as 236(92.2%) have never accompanied their wives/partners to clinics to seek family planning services. Also 242(94.5%) and 251(98%) of men, respectively, knew the importance of antenatal services and supervised delivery. However, only 114(44.5%) of men ever accompanied their wives/partners to seek skilled delivery services. Men’s involvement was hindered by barriers such as attitude of health workers, long waiting time and socio-cultural beliefs.

**Conclusion:**

The study revealed a gap between men’s awareness of the importance of maternal and safe motherhood services and their actual involvement in accessing these services with their female partners. There is a need to create a supportive environment that encourages men to be involved in maternal health services to help reduce maternal/neonatal morbidity and mortality.

## Background

The inclusion of men in maternal and safe motherhood services is increasingly recognised as an important determinant of women’s access to needed care in many low-income settings, including Ghana [1–5]. Men’s inclusion generally means encouraging men to become more involved and supportive of women’s needs, choices, and rights in sexual and reproductive health, and addressing men’s own sexual and reproductive health needs and behaviour [3, 4]. In this study, we defined men’s inclusion in maternal and safe motherhood services to include their involvement in family planning, ANC visits and supervised delivery.

It has been recognised that though maternal and newborn survival requires improvements in basic and comprehensive obstetric care coverage and quality, inclusion of men in maternal and safe motherhood services is required to increase the use of these services, eliminate delays in accessing care, and promote timely referral when problems arise [2–4]. In societies where patriarchal norms are dominant, men are often major decision-makers for the family, hence decisions around when, where and even if a woman should have access to healthcare often are made by men [1, 3, 4, 6, 7]. Within the context of maternal and safe motherhood services delivery, one study in Tanzania found that households headed by men were associated with more home deliveries [8]. Another study in Pakistan noted that high decision-making power by men was linked to low utilisation of antenatal and delivery care services [9]. A number of recent studies in Ghana have also indicated that men’s disapproval is a major barrier to women’s use of skilled maternal and newborn healthcare services [2, 5]. In some parts of the world, it has equally been noted that husband’s approval is an important determinant of access and use of maternal and safe motherhood services [10, 11].

The recognition that men in many low-income contexts play major roles as decision-makers in facilitating or discouraging access to needed maternal and safe motherhood services has led to calls for men to be actively involved in facilitating women’s access [1, 2, 5]. In fact, there is growing evidence that involving men in maternal and safe motherhood issues has benefits for timely access to needed services and could significantly influence health outcomes for women and children [10–18]. In Malawi [18] and Uganda [19], men’s inclusion in maternal health services is seen as a strategy for getting fast service for women during ANC visits, labour and delivery. In India, one study found that a woman whose husband was involved in her pregnancy was more likely to deliver in a health institution or in the presence of skilled medical personnel at home compared to women whose husbands were not involved [11].

Indeed, the inclusion of men in issues of maternal and safe motherhood is an important component of WHO’s recent recommendations on health promotion interventions for maternal and newborn health [20]. Consequently, an increasing number of programmatic efforts and initiatives to actively involve men in maternal and safe motherhood issues are being promoted in many sub-Saharan African contexts where the burden of maternal and child mortality is high and patriarchal norms and values dominant [3, 4]. At the same time, however, few empirical studies have been conducted to document the extent to which men are involved in issues of maternal health and safe motherhood [3, 4]. In Nigeria, one study found that only 27% of husbands ever followed their wives to the labour room [21].

A number of studies in Ghana have examined men’s attitudes and barriers to male involvement in maternal and child health in a rural context [3, 4, 22]. Some of the barriers to men’s involvement include lack of time, long waiting time at health facilities before care is received, and men’s perception that pregnancy and childbirth is women’s business. Similar barriers have been reported in Nepal [23], Kenya [24], Ethiopia [25] and Malawi [18]. While those studies from Ghana provide insight into men’s attitudes toward, and barriers to, male involvement in rural Ghana, these studies were exploratory qualitative studies and did not consider inner-city urban environments, where gender norms and attitudes may be different. This knowledge gap could potentially inhibit programmatic efforts to promote greater inclusion of men in maternal and safe motherhood services delivery in a wide range of contexts including inner-city communities in Ghana. The purpose of this study was to describe the level of men’s involvement in maternal and safe-motherhood services in inner-city communities in Ghana, and to assess the barriers to men’s involvement.

## Methods

### Study design

A descriptive cross-sectional quantitative study design was adopted for the study. House-hold survey using interviewer-administered questionnaire was used for data collection.

### Study site

The study was conducted in under-served inner-city communities in Chorkor, Accra. Located on the outskirts of Accra, Chorkor is one of the poorest low-income, indigenous communities in Accra. According to Ghana’s 2010 Population and Housing Census, the population of the community is 344,627, with an average growth rate of 6.0% per annum [26]. As a dangerously overpopulated area, Chorkor lacks basic infrastructure such as toilets, bath houses, drains, and roads [27]. Houses are generally made of brick, mud and plywood [27]. Educational achievements are very low, and most community members engage in fishing, fish mongering, and fish smoking as their primary occupation [27]. The area frequently experiences communicable disease outbreaks such as cholera, which are mostly related to unhygienic practices. The lack of proper sewage systems and drains causes waste water to run from home to home as children defecate along drains in the community [27]. Additionally, most trash end up on the beach and ocean, which is then later pulled back ashore by fishing nets.

### Study population

The study population was married men who were aged 18 years and above whose wife/partner was pregnant and was in her third trimester or had a child (ren) less than or equal to five years old.

### Sampling procedure

A multi-stage sampling procedure was followed to select houses, households and respondents. First, a simple random sampling technique was employed to select the number of houses (i.e. 256) to be included in the study. There is data from the Ghana Statistical Service specifying house numbers within the Chorkor community. This information was compiled during the 2010 Population and Housing Census. Based on this information, we used an electronic or computer-based number generator to randomly select 256 houses for the study. Second, where any of the randomly selected houses had only one household (defined as a group of people sharing a common dwelling and eating arrangement) with only one male adult who met the inclusion criteria (i.e. aged 18+ and had a wife/partner who was pregnant and was in her third trimester or at least 7 months pregnant or had a child less than or equal to five years old), that household was selected. However, where there were more than one households in a selected house with at least one adult male who met the inclusion criteria, a simple random sampling technique was used to select one household. This was done by giving each household a number (e.g. 1–3). These numbers were then written on pieces of papers, foldered and kept in a bowl. At random, one of the folded pieces of paper was selected and the household corresponding to the selected number was included in the study. Third, where there were more than one adult men in the selected household who met the inclusion criteria, a simple random sampling technique was also used to select only one man. This was again done by giving each man a number (e.g. 1–4). These numbers were again written on pieces of papers, foldered and kept in a bowl. At random, one of the folded pieces of papers was selected and the man corresponding to the selected number was included in the study. Finally, where there were no households with men in the selected house who met the inclusion criteria, the house was replaced with the next house.

### Data collection procedure

A structured, interviewer-administered questionnaire was used to collect the data on socio-demographic variables such as age, education level, income, religion and employment status, participation in family planning, accompanying wives on ANC visit, birth process (labour and delivery). Five field assistants were recruited to collect the data using the house-to-house survey.

An interviewer administered questionnaire was used to collect data. The questionnaire was administered face-to-face by one of the researchers and research assistants.

Prior to the actual data collection, the survey instrument was pre-tested in Kole Gono, an adjourning inner-city community with characteristics very similar to Chorkor. This enabled us to detect improperly formulated questions as well as gauge the amount of time needed for each interview. All errors noted during the pre-test were corrected and the survey instrument fully revised before use in the final data collection.

### Data analysis

Data collected were screened by the researchers to check for completeness and consistency of responses. The responses were coded and entered into the Statistical Package for Social Sciences (SPSS) version 20. Results were presented as frequency and percentage distributions.

In addition to socio-demographic variables, a number of variables were also defined and assessed. Men’s inclusion in family planning (FP) was assessed by asking respondents to indicate whether they have ever accompanied their wife/partner to an FP Clinic. Men’s understanding of FP was also assessed by asking respondents to indicate the most appropriate meaning of FP from a list of possible responses. Other variables included whether men discussed use of FP methods with their wife/partner. In relation to inclusion of men in ANC services, respondents were asked whether they have accompanied their wife/partner (at least once) to ANC clinic during their last/current pregnancy. Respondents’ knowledge on the importance of ANC was also evaluated by asking respondents to indicate the most appropriate function of ANC from a list of possible responses. Other variables included whether men knew the health care services provided to pregnant women at ANC, whether men help their wife/partner in her household chores in pregnancy, whether men save money for safe delivery of their wife/partner, and whether men arranged for their wives’ transport to ANC-clinics. Finally, a number of variables were also assessed in relation to supervised delivery. Specifically, men’s inclusion was assessed by asking whether a man accompanied his wife/partner for supervised delivery during their last pregnancy, whether a man will accompany wife/partner to a health facility to deliver in current/future pregnancy, and men’s knowledge of the importance of skilled delivery.

## Results

A total of 256 questionnaires were administered. All of them were fully completed and returned, giving a response rate of 100%.

### Socio-demographic characteristics of respondents

Table 1 shows the socio-demographic characteristics of respondents. The mean age of respondents was 35.7 years with a standard deviation of 9.7 years. More than a quarter 102(39.8%) of the men were aged between 25 and 34 years, followed by men in the age group of 35–44 years 78(30.5%). With respect to education, 77(30.1%) had no formal education at all. Furthermore, 115(45%) of their wives/partners also had no education at all. In relation to occupation, majority of the men were fishermen 125(58.2%).

**Table 1:** Socio-demographic characteristics of respondents

Variable	Frequency, *n* = 256	Percentage (%)
Age of man (years)		
19–24	25	9.8
25–34	102	39.8
35–44	78	30.5
45–54	40	15.6
55–64	10	3.9
65+	1	0.4
Educational level of man		
None	77	30.1
Primary	55	21.5
Junior High/middle School	83	32.4
Senior High School	30	11.7
Tertiary	11	4.3
Educational level of wife/partner		
None	115	44.9
Primary	38	14.8
Junior High/middle School	80	31.3
Senior High School	23	9.0
Tertiary	0	0.0
Marital status		
Currently married	215	84.0
Co-habitation	28	10.9
Divorced/separated	13	5.1
Number of wives/partners		
One	200	78.1
Two	44	17.2
Three	12	4.7
Currently staying with wife/partner		
Yes	149	58.2
No	107	41.8
Occupation		
Fishing	125	48.8
Trading/business	56	21.9
Government salary worker	27	10.5
Private salary worker	47	18.4
Unemployed	1	0.4
Religion		
Christianity	223	87.1
Islam	6	2.3
Traditional	27	10.5
Number of children		
0–4	180	70.3
5–9	69	27.0
10–13	7	2.7
Wife/partner has NHIS card		
Yes	169	66.0
No	84	32.8
Don’t know	3	1.2

### Men’s involvement in family planning (FP)

Table 2 displays results on male involvement in family planning. Almost all the men knew something in relation to the meaning of family planning (FP) except one. Of 255 men who knew the meaning of FP, 149(58.4%) said it was about limiting the number of children, followed by 89(34.9%) who said it was the spacing of birth intervals. Only 1(0.4%) said it was a means to destroy marriages. In terms of men’s involvement, only 17(6.6%) have ever accompanied their wives/partners to the FP clinic. Similarly, majority of the respondents 174(68%) said they did not discuss the use of FP methods with their wives/partners. On current use of FP methods, only 54(21.1%) of the respondents said their wives/partners were using some methods. Only 16 (6.3%) did not know if their wives/partners were using FP methods.

**Table 2:** Men’s Inclusion in Family Planning activities

Variable	Frequency	Percentage
Ever accompanied wife/partner to FP Clinic		
Yes	17	6.6
No	236	92.2
Participants’ understanding of FP (*n* = 255)		
Spacing the birth intervals of children	89	34.9
Limiting the number of children	149	58.4
A means by ‘the white man’ to reduce the population of Africans	9	3.5
It is a means to give power to women	7	2.7
It is a means to destroy marriages	1	0.4
Discuss use of FP methods with wife/partner?		
Yes	79	30.8
No	174	68.0
Not applicable	3	1.2
Wife/partner currently using any FP methods?		
Yes	54	21.1
No	186	72.7
Don’t know	16	6.3

### Men’s inclusion in antenatal care (ANC)

Table 3 also illustrates men’s involvement in ANC. The results show that 209(81.6%) of the men have never accompanied their wives/partners to visit the ANC clinic. Further analysis of the data (not shown here) showed that of the 47(8.4%) men who have ever accompanied their wives/partners to ANC, 19(40.4%) did so only once, 24(51.1%) went twice and only 4(8.5%) went at least four times. Similarly, out of the 209 men who never accompanied their wives to ANC-clinics, 134 (64.1%) and 47 (22.5%), respectively, cited ANC as being the responsibility of the woman, and they being busy with work as their reasons. Only 15 (7.2%) and 13(6.2%), respectively, cited shyness, and wife feeling uncomfortable going to the ANC with them, as their reasons.

**Table 3:** Men Inclusion in Antenatal Care activities

Variable	Frequency	Percentage
Wife/partner attended (at least once) ANC during last/current pregnancy		
Yes	245	95.7
No	2	0.8
Don’t know	9	3.5
Accompany wife/partner (at least once) to ANC clinic during last/current pregnancy		
Yes	47	8.4
No	209	81.6
Respondents’ knowledge on importance of ANC (*n* = 242)		
Ensures safe delivery	118	48.8
Identify and prevent pregnancy complications	20	8.3
Monitors the growth of the foetus	61	25.2
Ensures good health and proper care of woman	43	17.8
Know health care services provided to pregnant women at ANC (n = 242)		
Yes	62	25.6
No	180	74.4
Helps wife in her household chores in pregnancy		
Yes	135	52.7
No	121	47.3
Household chores in which man assists wife (*n* = 135)?		
Washing clothes/dishes	114	84.4
Cooking	13	9.6
Bathing older children	8	5.9
Save money for safe delivery of wife/partner?		
Yes	254	99.2
No	2	0.8
Arrange for transportation for wife to go to health facility for ANC		
Yes	216	84.4
No	40	15.6

Notwithstanding the relatively low involvement of men, most of them had some understanding of the importance of ANC. Specifically, 118(48.8%) of the respondents said that ANC may ensure safe delivery; 61(25.2%) said it monitors the growth of the foetus; 43(17.8%) said it ensures good health and proper care of women; and 20(8.3%) said ANC may identify and prevent pregnancy complications. Also, 245(95.7%) of the men reported that their wives/partners attended ANC at least once during their current or most recent pregnancy. However, 180(74.4%) men did not know the kind of healthcare services that was provided to pregnant women, including their wives/partners, at ANC clinics.

Other forms of involvement, including saving money towards safe delivery and arranging transport for their wife/partner to visit ANC clinic, were also assessed. Most respondents 254(99.2%) saved money for safe delivery of their wives/partners (Table 3). Also 216(84.4%) of the men reported to arrange transport for their wives to visit ANC clinics for check-up.

### Men’s inclusion in supervised delivery

Table 4 shows that more than half 142(55.5%) of the respondents did not accompany their wife/partner for delivery in a health facility during her last pregnancy. When asked whether they will accompany their wife/partner to the health facility for delivery in the future, only 56(21. 9%) men answered yes.

**Table 4:** Men inclusion in supervised delivery activities

Variable	Frequency	Percentage
Accompanied wife/partner for supervised delivery during last pregnancy		
Yes	114	44.5
No	142	55.5
Respondents’ knowledge on importance of supervised delivery		
Woman gets the best of care	32	12.7
Avoids complications	45	17.9
It is safer for mother and child	174	69.3
Will accompany wife/partner to health facility to deliver in current/future pregnancy		
Yes	56	21.9
No	200	78.1
Decision-maker on place of delivery		
Husband	80	31.3
Wife	2	0.8
Both husband and wife	79	30.9
Mother-in-law	95	37.1

Despite the relatively low levels of men’s involvement in supervised delivery, most of them knew that supervised delivery was important. Majority 174(69.3%) said supervised delivery is safer for mother and child while 45(17.9%) said it helped to avoid and/ or address complications during delivery. More than a quarter of the men (95, 37.1%) said their mothers-in-law decided on the place of delivery, while 79(30.9%) indicated that both they and their wives/partners jointly chose the place of delivery. Only 2(0.8%) of the men said their wives/partners made the decision alone.

### Barriers to men’s inclusion in safe motherhood services

Most respondents (220, 85.9%) reported that long waiting time at health facilities before care is given was a barrier to their involvement in safe motherhood services. Other important barriers included poor attitude of health workers (197, 77%), lack of time to accompany spouses (214, 83.6%), and the fact that some safe motherhood services focused exclusively on women (180, 70.3%). However, majority of the respondents disagreed that distance to health facilities (148, 57.8%), cost of services (144, 56%), lack of information/knowledge about maternal and safe motherhood services (133, 52%), shyness to take part in maternal and motherhood services (180, 70.3%), and dissatisfaction with services in health facilities (157, 61.3%) were barriers to their involvement (see Table 5).

**Table 5:** Barriers to men’s inclusion in safe motherhood services

Variable/Statement	Agree, n (%)	Disagree, n %)
Long waiting time in health facilities	220 (85.9)	36 (14.1)
Distance to health facilities	108 (42.2)	148 (57.8)
Attitude of health workers	197 (77.0)	59 (23.0)
Cost of services	112 (44.0)	144 (56.0)
Lack of time to accompany spouse for the services	214 (83.6)	42 (16.4)
Cultural beliefs	158 (61.7)	98 (38.3)
Religious beliefs	42 (16.4)	214 (83.6)
Lack of information/knowledge on safe motherhood/ reproductive health service	123 (48.0)	133 (52.0)
Some safe motherhood services are mainly focused on women only	180 (70.3)	76 (29.7)
Shyness to take part in safe motherhood services as a man	76 (29.7)	180 (70.3)
Dissatisfaction with the safe motherhood services rendered in health facilities	99 (38.7)	157 (61.3)

## Discussion

### Main results

This study is one of the few to have focused on the inclusion of men in maternal and safe motherhood services in an inner-city in urban Ghana. The study revealed that most of the men correctly recognised the benefits of FP, ANC and skilled delivery care. About 35% and 58% of men, respectively, understood FP to mean only limiting the number of desired children by a couple, and birth spacing between children. This is similar to findings from a study in Nigeria, where 38% and 35% of men cited control of family size and birth spacing respectively as the importance of FP [21]. Similarly, and in relation to ANC, most of the men recognised the importance of ANC during pregnancy. This is consistent with findings from Papua New Guinea where 84% of men considered ANC as important, and knew the benefits of ANC during pregnancy [28]. Furthermore, the study also revealed that most of the men (174, 69.3%) knew that supervised delivery could ensure safer birth for mother and survival of the child.

Despite men’s recognition of the importance of the three domains of maternal and safe motherhood services examined in the study, majority of them were not actively involved in a number of key FP, ANC and supervised delivery services. While the relatively high level of men’s recognition of the functions of FP could be attributed to extensive media publicity and education by health workers and partners in the country, only about 17(7%) men have ever accompanied their wives/partners to an FP clinic. This proportion is far lower than results from a Nigerian study in which 29% of men accompanied their wives to FP clinics [21]. This relatively low level of men’s involvement in FP is importantly further reflected in the fact that 174(68%) did not discuss issues of FP with their wives/partners. The revelation that more than two-thirds of the men do not discuss use of FP with their spouses is clearly concerning as spousal communication has been found to improve use of FP services among couples. Lack of spousal communication about the use of any FP methods by one’s partner could easily lead to suspicion, petty quarrels and gender-based violence. Perhaps this is part of the reasons why only 21.1% of the wives/partners currently used any FP methods.

But it is not only in the context of FP that male involvement is low. Results relating to men’s inclusion in ANC services also suggest that although some men are actively engaged in saving money for safe delivery as well as arranging transport for visit to ANC clinics, the majority had never accompanied their wives/partners to visit the ANC clinic. This relatively low involvement of men was reflected in men’s knowledge of ANC services received at ANC clinics. As regards supervised delivery, a similarly low level of men’s involvement was observed. What is even worrying is the observation that only 22% of the men said they would accompany their wife/partner to a health facility for delivery in future. These results are however not surprising: previous studies in the Jinja District of Uganda [29] and a Nigerian community [21] have, respectively, found that only 43.4% and 27% of men, accompanied their wives to the health facility for delivery during her most recent delivery.

The relatively low involvement of men was framed by several contextual, social, economic and gender-related factors. For instance, 220(86%) men reported long waiting time at health facilities before care is given as a barrier to their involvement in safe motherhood services. In Ethiopia [25], Uganda [29], and Ghana [3, 4], similar results have been documented: waiting time of more than 30 min in a hospital prevented men from accompanying their wives to health facilities. Time is particularly important in the context of Chorkor where economic and social living conditions are harsh and where men – who are usually the bread winners – have to spend long hours at sea fishing. This suggests a need for interventions such as expansion of FP and ANC service delivery points as well as individual booking systems to reduce the amount of time women have to spend before care is received. This may encourage men to actively support their wives/partners to receive needed FP, ANC and supervised delivery services.

Another factor that could explain men’s relatively low involvement in maternal and safe motherhood services in Chorkor relates to the attitude of health workers, which more than three-quarters expressed dissatisfaction with. Attitude of health workers as a barrier has been reported in several studies [1–5, 7–13]. Attitude of health workers has a particularly huge influence on health care services utilization more generally and men’s involvement in maternal and safe motherhood services precisely because clients expect respect from their health care providers. When clients are scorned or disrespected by their healthcare providers, they may never want to visit or return to such service providers. The issue of respect is particularly critical in a largely patriarchal context like Ghana where men expect respect from women. If men are to be actively involved in FP, ANC and supervised delivery services in contexts such as Ghana, there is a need for service providers to be trained in basic interpersonal communication skills so as to enable them provide more supportive and respectful services to couples.

Cultural beliefs have also been revealed in this study to act as barriers to male involvement. In some Ghanaian and African societies, men who engage in some form of safe motherhood services are mocked by their peers. They say such men are ‘weak’ and controlled by their wives, hence men who are unable to withstand the name calling and mockery are forced to withdraw [3, 4]. As shown earlier in relation to FP, many men were of the belief that FP is the sole responsibility of women so that men who accompany their wives to the FP clinic are seen as ‘weak’ and kowtowing to their wives. This is more likely to be the case given that patriarchal norms are very entrenched in many Ghanaian communities [3, 4]. Indeed, studies have found that men do not accompany their wives to FP clinics precisely because of perceptions that FP is women’s business [24].

But many men also felt alienated by some of the services that are being provided to pregnant women. For instance, 180(70%) of the men reported that some safe motherhood services are focused on women only which do not encourage them to fully take part. This would suggest a need for both continuous community education and couple counselling to correct misconceptions and re-inforce the fact that maternal and safe motherhood services like FP are not solely the business of women. At the same time, there is a need to fashion out more innovative strategies to engage men as active partners beyond the provision of money, arranging transport or assisting in household chores. For example, invitation cards could be used to invite men to routine ANC classes and pregnancy schools to enhance men’s understanding of maternal and safe motherhood issues.

### Limitations of the study

Although the results of this study have shown the extent to which men are involved in maternal and safe motherhood issues, the study has certain limitations. Since this was a cross-sectional study, it was impossible to assess cause and effect relationships. Related to this design limitation is the fact that the study was descriptive and did not examine association between socio-demographic or other factors and men’s involvement in FP, ANC and supervised delivery services. Future studies should include this. Again, and regarding men’s understanding of FP, ANC and supervised delivery, a respondent was asked to choose only one answer they considered the most appropriate from a list of possible answers. While this ensured analytical precision by showing the exact understanding of each respondent, we acknowledge that there may exist two or more appropriate answers. In other words, multiple responses could have been allowed. Finally, the study was only conducted in one out of several low-income inner-city communities in Accra, and also involved relatively small sample size. Therefore, the limitation of generalising these results is acknowledged.

## Conclusion

This study has shown that despite men’s recognition of the importance of FP, ANC and supervised delivery services, their involvement in the delivery of FP, ANC and supervised delivery services to their wives/partners is relatively low. Among the factors that militate against men’s active involvement are the attitude of health workers, long waiting time in health facilities and socio-cultural beliefs. Considering the influence and power of men as decision makers in the utilization of safe motherhood services, there is a need to create a supportive community- and health facility-level environment that encourages men to become actively involved. It is critical to include effective ways of delivering education messages to men by using role models and peers to engage them. Attempts should be made to ensure that FP, ANC and supervised delivery services are organized and delivered in a manner that is more male-friendly, and with appropriate health campaigns to change attitudes and beliefs of men towards becoming more actively engaged in supporting their wives/partners to receive skilled and safe motherhood services.

## Abbreviations

ANC: Antenatal care
FP: Family planning
NHIS: National Health Insurance Scheme
SPSS: Statistical Package for Social Sciences
WHO: World Health Organization
